# Nicotine regulates autophagy of human periodontal ligament cells through α7 nAchR that promotes secretion of inflammatory factors IL-1β and IL-8

**DOI:** 10.1186/s12903-021-01894-5

**Published:** 2021-11-03

**Authors:** Yang Du, Kuan Yang, Zhifei Zhou, Lizheng Wu, Lulu Wang, Yujiang Chen, Xin Ge, Xiaojing Wang

**Affiliations:** 1 State Key Laboratory of Military Stomatology & National Clinical Research Center for Oral Diseases & Shaanxi Key Laboratory of Stomatology, Department of Pediatric Dentistry, School of Stomatology, The Fourth Military Medical University, No.145 West Changle Road, Xi’an, 710032 Shaanxi China; 2 Department of Orthodontics, College of Stomatology, Xi’an Jiaotong University, Xi’an, China; 3 Department of Stomatology, The General Hospital of Tibetan Military Region, Lhasa, China; 4 Department of Stomatology, Characteristic Medical Center of People’s Armed Police Force, Tianjin, China

**Keywords:** Nicotine, Autophagy, α7 nAchR, Periodontitis

## Abstract

**Background:**

Nicotine is an important risk factor and the main toxic component associated with periodontitis. However, the mechanism of nicotine induced periodontitis is not clear. To investigated the mechanism through which nicotine regulates autophagy of human periodontal ligament cells (hPDLCs) through the alpha7 nicotinic acetylcholine receptor (α7 nAChR) and how autophagy further regulates the release of IL-1β and IL-8 secretion in hPDLCs.

**Methods:**

HPDLCs were obtained from root of extracted teeth and pre-incubated in alpha-bungarotoxin (α-BTX) or 3-Methyladenine (3-MA), followed by culturing in nicotine. We used a variety of experimental detection techniques including western blotting, immunofluorescence, enzyme-linked immunosorbent assay (ELISA), transmission electron microscopy (TEM) and RT-qPCR to assess the expression of the LC3 protein, autolysosome, and release of IL-1β and IL-8 from hPDLCs.

**Results:**

Western blots, immunofluorescence and TEM results found that the nicotine significantly increased the autophagy expression in hPDLCs that was time and concentration dependent and reversed by α-BTX treatment (*p* < 0.05). RT-qPCR and ELISA results revealed a noticeable rise in the release of inflammatory factors IL-1β and IL-8 from hPDLCs in response to nicotine. RT-qPCR and ELISA results showed that nicotine can significantly up-regulate the release of inflammatory factors IL-1β and IL-8 in hPDLCs, and this effect can be inhibited by 3-MA (*p* < 0.05).

**Conclusions:**

Nicotine regulated autophagy of hPDLCs through α7 nAChR and in turn the regulation of the release of inflammatory factors 1L-1β and 1L-8 by hPDLCs.

**Supplementary Information:**

The online version contains supplementary material available at 10.1186/s12903-021-01894-5.

## Background

Periodontitis is a chronic progressive infectious disease with a high prevalence of 45–50% overall, with the most severe form affecting 11.2% of the world's population, being the sixth most common human disease [1, 2]. Tobacco smoking has been recognized as one of main risk factors for the occurrence and development of periodontitis [3]. Nicotine is one of the most toxic substances in tobacco [4]. It can cause pathological changes in human periodontal tissues, promote alveolar bone resorption, and ultimately lead to tooth loss [5].

Alpha7 nicotinic acetylcholine receptor (α7 nAChR) is a predominant subunit of nicotinic acetylcholine receptors (nAChRs), as well as a potent target of the nicotine binding receptor [6]. Previously, we had demonstrated the functional expression of α7 nAChR in periodontal ligament (PDL) tissues and human periodontal ligament cells (hPDLCs). Nicotine can enhance the expression of α7 nAChR in PDL tissues and hPDLCs, activate inflammation-related signaling pathways, and further regulate the secretion of inflammatory cytokines such as IL-1β and IL-8 [6–8].

Autophagy is an essential cellular mechanism which plays “housekeeping” role in normal physiological processes including removing of long lived, aggregated and misfolded proteins, clearing damaged organelles, growth regulation and aging [9].

Autophagy is a dynamic multi-step process that involves the formation of autophagosomes, fusion of the autophagosome with the lysosome to form the autolysosome, and finally the degradation of the contents in the autolysosome [10, 11].The measurement of fluorescently labeled LC3 puncta and autolysosomes in cells can be used as a method to quantify autophagy [10].

Autophagy could influence the pathogenesis of various inflammatory disorders [12]. Recent studies have shown evidences of autophagy in periodontal tissue, which may be involved in periodontitis [9]. For instance, the expression of autophagy-related factors was elevated in periodontitis [13]. In addition, autophagy also exerted multiple effects on hPDLCs in different conditions [14]. In current years, the interaction between nicotine and autophagy has been discussed [15]. Additionally, nicotine regulated the autophagy process via nAChRs in other cell types [16]. So, we assume that autophagy may also participate in smoking-related periodontitis via the regulation of inflammation-related signaling that leads to inflammatory disorders and periodontal tissue damage.

Given this, the aim of the study was to investigate the mechanism which nicotine regulated autophagy of hPDLCs through the α7 nAChR and how autophagy further regulated the secretion of inflammatory factor IL-1β and IL-8 in hPDLCs.

## Methods

### Isolation and culturing of hPDLCs

The current study included caries free and periodontally healthy premolars (n = 14) that were extracted due to the orthodontic reasons from young patients (12–16 years). We received approval from the institutional Ethical Review Board at the School of Stomatology, the Fourth Military Medical University, China. The children’s parents/guardians were informed about the purpose of the study and inclusion of the extracted teeth in the research and provided informed consent in written format. The extracted teeth were cleaned and stored in the DMEM (Hyclone) containing 15% fetal calf serum (FCS) (Hyclone) and antibiotics (Invitrogen). Isolation and culturing of hPDLCs was performed as described previously [17]. In order to avoid contamination from the gingival and pulpal tissues, PDL tissues were excised from the middle third of the root using a sharp scalpel. PDL tissues were seeded in a six-well plate (Hyclone) and cultured at 37 °C in a humidified atmosphere with 95% air and 5% CO_2_. The medium was changed every 3 days. After achieving the confluence, the cell layers were sub-cultured and used for further experiments.

### Treatment with nicotine, α-BTX and 3-MA

Based on previous studies [8, 18], this study designed that nicotine (Sigma) was administrated to hPDLCs either at variable concentrations (10^−4^, 10^−5^, 10^−6^, 10^−7^ mol/L) for 12 h, or a fixed concentration (10^−5^ mol/L) for a variable period of time (3, 6, 9, 12 and 24 h). In order to prove that nicotine-induced autophagy of hPDLCs was mediated by α7 nAChR, according to previous studies [6, 8], we pretreated hPDLCs with α-BTX (10^−8^ mol/L, α7 nAChR specific receptor antagonist) (Tocris Bioscience), for 30 min before nicotine (10^−5^ mol/L, 12 h) stimulation. To further examine whether autophagy played a role in the secretion of inflammatory factors in hPDLCs, according to the literature report [19, 20], we pretreated hPDLCs with 3-methyladenine (3-MA) (10^−3^ mol/L, a PI3K inhibitor that effectively blocks autophagy) (Sigma), for 30 min before nicotine (10^−5^ mol/L, 12 h) stimulation, the expression of autophagy and the secretion of IL-1β and IL-8 were detected.

### Protein isolation and Western blot

Protein extraction was performed as described previously [21]. To prepare the total cell lysate, the cells (density, 1 × 10^6^ cells/dish) were seeded and then treated with nicotine, α-BTX, or 3-MA various agents, cell proteins were isolated using the Nuclear Extract Kit (Sangon Biotech). The BCA kit (Thermo) was used to calculate protein concentration in the solution. Proteins (40 µg/lane) were isolated with SDS-PAGE and then transferred to a PVDF membrane (Millipore). Following blocking with 5% skimmed milk dissolved in Tris-buffered saline containing 0.1% Tween-20 at 37 °C for 1 h, the membranes were probed with primary antibodies against: LC3B, Beclin-1, GAPDH (Cell Signaling Technology) at 4 °C overnight. After three washes, membranes were incubated with secondary antibody for 1 h at 37 °C. An Infrared Imaging System (Odyssey) was used to assess blots. Representative results from one of three independent experiments were shown.

### Immunofluorescence

For immunofluorescence, the cells were fixed in 4% paraformaldehyde for 30 min, washed using the PBS (Hyclone), permeabilized at room temperature using the 0.5% Triton X-100 and blocked with BSA (Sigma) for 2 h. Cells were then incubated with LC3B primary antibody (Cell Signaling Technology) at the 1:200 dilution. Cells were washed using PBS and incubated with the FITC-labeled secondary antibody (1:1000). Cell nuclei stained by the Hoechst (Sigma) were analyzed using a fluorescence microscope (Olympus FV1000). In this study, the method of immunofluorescence point counting was described previously [22]. Representative results from one of three independent experiments are shown.

### Transmission electron microscope assays (TEM)

The TEM samples as prepared as were described previously [23]. Briefly, cells were fixed using the 2.5% glutaraldehyde in 0.1 mol/L cacodylate buffer for 30 min. Representative ultra-thin sections were analyzed using standard TEM methods. Representative results from one of three independent experiments are shown.

### Real-time quantitative polymerase chain reaction (RT-qPCR)

In order to detect IL-1β and IL-8 expression, total RNA was isolated and RT-qPCR assay were performed as described previously [24]. Briefly, we used TRIZOL reagent (Takara) to extract the total RNA according to the manufacturer’s instructions and the PrimeScript RT reagent Kit (Takara) to synthesize the cDNA. All experiments were conducted in triplicate using Mastercyclerep realplex (Eppendorf AG) and GAPDH as an internal standard. The following primer sequences were used: human IL-1β forward: 5'-ATGATGGCTTATTACAGTGGCAA-3' and reverse, 5'-GTCGGAGATTCGTAGCTGGA-3'; human IL-8 forward: 5'-TTGCCAAGGAGTGCTAAAGAA-3' and reverse, 5'-GCCCTCTTCAAAAACTTCTCC-3'; GAPDH forward 5'-ACCCACTCCTCCACCTTTG-3'' and reverse, 5'-ATCTTGTGCTCTTGCTGGG-3'. Gene expression was calculated using the 2^−ΔΔCq^ method [25]. Experiments were performed in triplicate.

### Enzyme linked immunosorbent assay (ELISA)

Cell culture supernatants were analyzed using ELISA for detecting IL-1β and IL-8 release as described previously [26]. The highly sensitive ELISA kits from R&D systems (Minneapolis) were used to analyze IL-1β and IL-8 concentrations and normalized to the number of cells. The supernatants were thawed once and all assays were run at the same time. Each set of experiments was performed in triplicate.

### Statistical analysis

All data from triplicate experiments was analyzed using the SPSS software (Version 21, IBM) and presented as mean ± standard deviation (SD). Student's t-test was used to compare the control and treatment groups, and multiple comparisons were performed using one-way ANOVA. *P* < 0.05 was considered to indicate a statistically significant difference.

## Results

### Nicotine increased LC3 fluorescence puncta and autolysosomes in hPDLCs in vivo

**Fig. 1:**
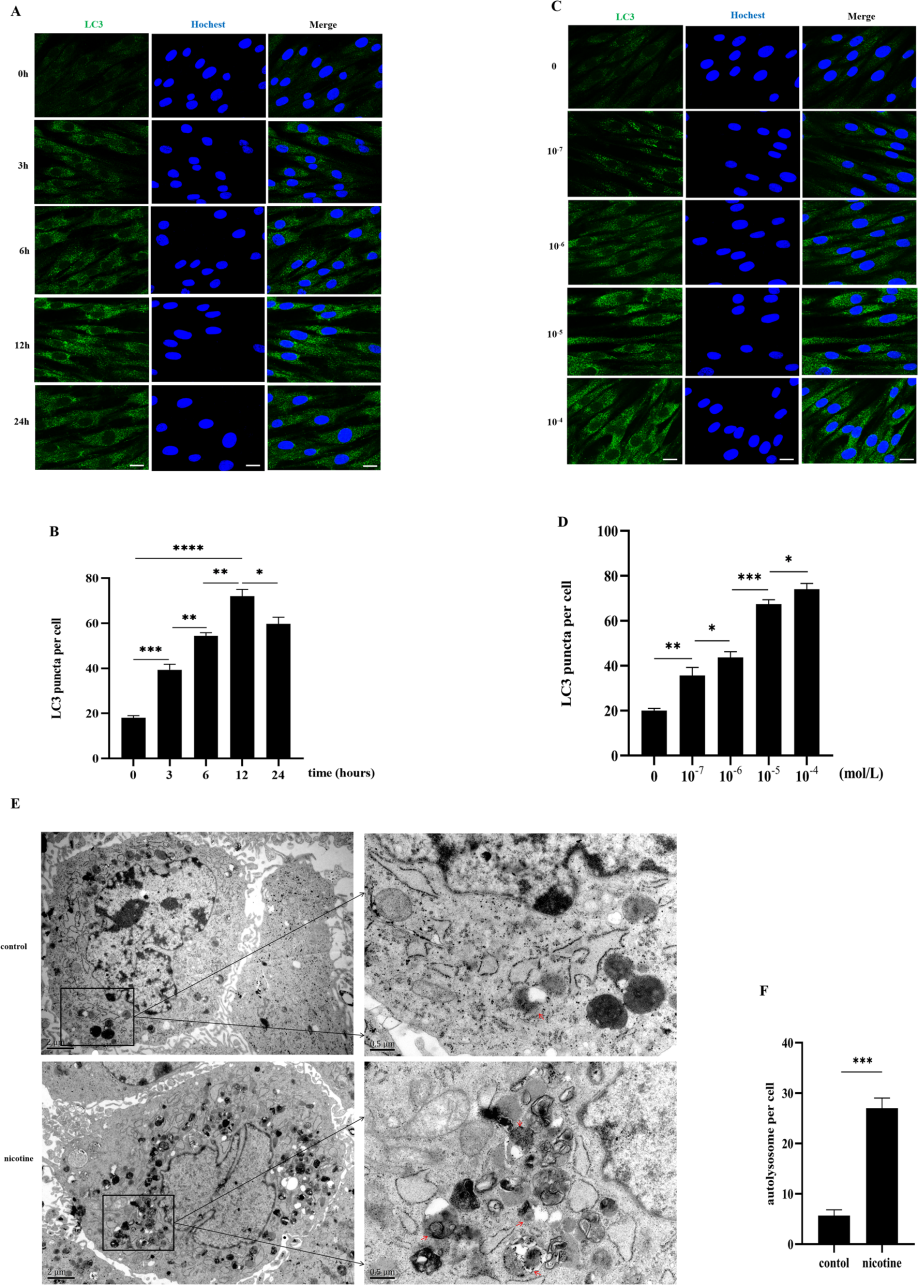
Nicotine increased LC3 fluorescence puncta and autolysosomes in hPDLCs. **A** LC3 fluorescence puncta in hPDLCs after nicotine (10^−5^ mol/L) treatment at time (0, 3, 6, 12, 24 h) as determined by immunofluorescence using confocal laser scanning microscopy. Scale bars represent 100 µm. **B** Bar graph showing the number of LC3 fluorescence puncta per cell for at least 10 cells per group. **C** LC3 fluorescence puncta in hPDLCs after nicotine (10^−4^, 10^−5^, 10^−6^, 10^−7^ mol/L) treatment at 12 h as determined by immunofluorescence using confocal laser scanning microscopy. Scale bars represent 100 µm. **D** Bar graph showing the number of LC3 fluorescence puncta per cell for at least 10 cells per group. **E** The formation of autolysosomes in hPDLCs induced by nicotine (10^−5^ mol/L, 12 h) was observed by TEM. Magnification, 40,000 × . Scale bar, 0.5 µm. The red arrows represent autolysosomes. **F** Quantitative analysis of autolysosomes in one cell, at least 3 cells in each group for statistics. (*p < 0.05, **p < 0.005, ***p < 0.001, ****p < 0.0001)

According to immunofluorescence results (Fig. 1A, B), nicotine (10^−5^ mol/L) treatment of hPDLCs for 3 h significantly increased LC3 fluorescence puncta (*p* < 0.001). The increase peaked at 12 h of nicotine treatment (*p* < 0.0001) and decreased after that (*p* < 0.05). Using a nicotine application length to hPDLCs for 12 h, we observed dose-dependent increases in LC3 fluorescence puncta around the nucleus (Fig. 1C, D). In our previous study, it was found that the nicotine concentration was greater than 10^−5^ mol/L, and the activity of hPDLCs was significantly reduced [18]. As nicotine (10^−5^ mol/L) treatment for 12 h significantly enhanced autophagy in hPDLCs, these conditions were chosen for subsequent experiments.

In subsequent experiments, we verified the formation of autolysosomes in hPDLCs using TEM under the action of nicotine at this concentration (10^−5^ mol/L) and time (12 h). TEM results (Fig. 1E, F) showed that there were more autolysosomes in nicotine-treated hPDLCs than in the control group (*p* < 0.001). The results of this part indicate that nicotine can promote the formation of autophagy in hPDLCs.

### Effects of nicotine on hPDLCs autophagy were mediated through α7 nAChR

**Fig. 2:**
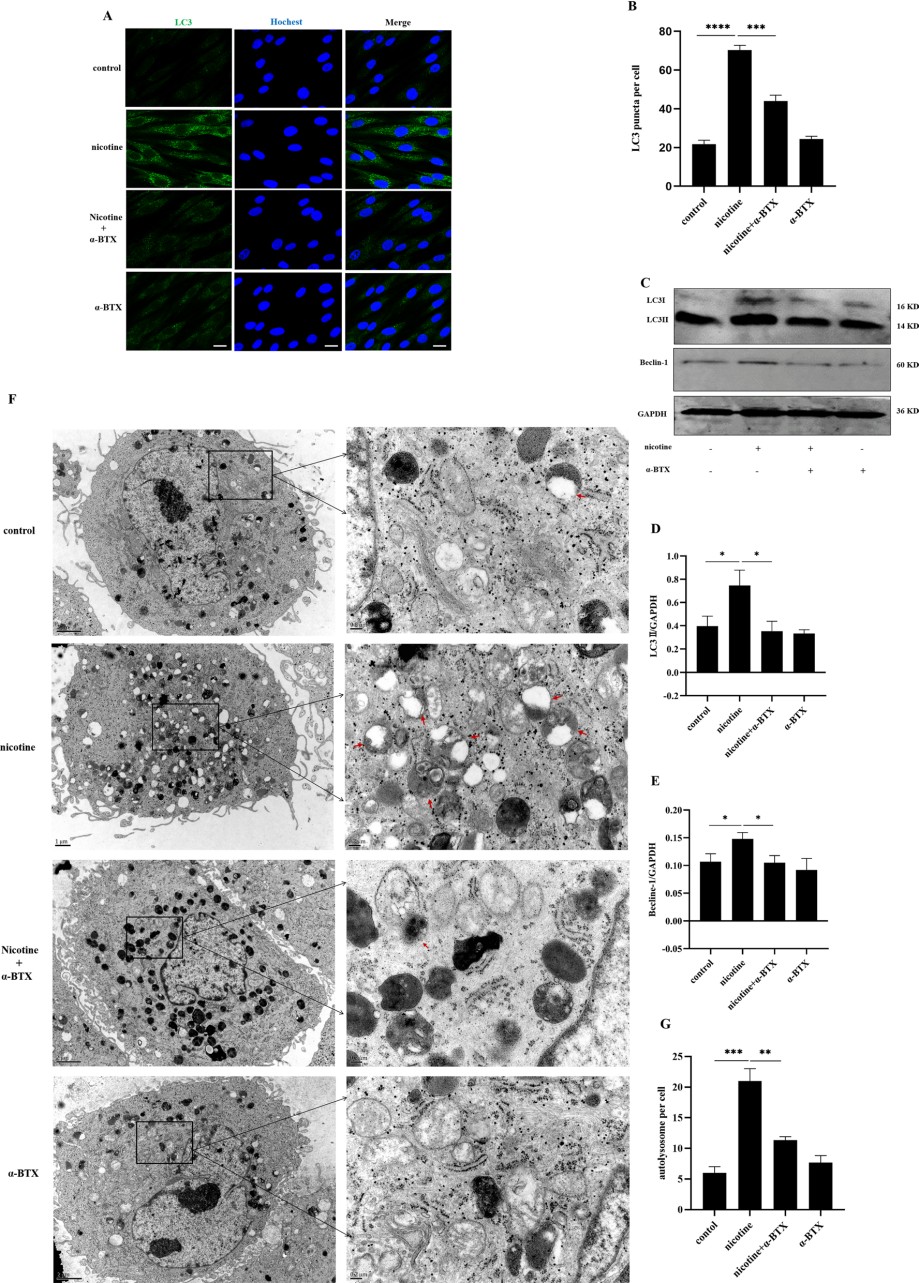
Effects of nicotine and/or α-BTX on the expression of autophagy protein and autolysosomes in hPDLCs. **A** LC3 fluorescence puncta in hPDLCs after nicotine and/or α-BTX as determined by immunofluorescence using confocal laser scanning microscopy. Scale bars represent 100 µm; **B** Bar graph showing the number of LC3 fluorescence puncta per cell for at least 10 cells per group; **C** LC3II and Beclin-1 protein expression in hPDLCs assessed by Western blot analysis; **D** LC3II protein quantitative analysis. Data were expressed as mean ± SD from at least three independent experiments; **E** Beclin-1 protein quantitative analysis. Data were expressed as mean ± SD from at least three independent experiments; **F** TEM was used to evaluate autophagy induced by nicotine (10^−5^ mol/L, 12 h) and/or α-BTX (10^−8^ mol/L, 12.5 h). Magnification, 40,000×. Scale bar, 0.5 µm. The red arrows represent autolysosomes; **G** Quantitative analysis of autolysosomes in one cell, at least 3 cells in each group for statistics (*p < 0.05, **p < 0.005, ***p < 0.001, ****p < 0.0001)

In order to explore the role of α7 nAChR in nicotine-induced autophagy of hPDLCs, we pretreated hPDLCs with α-BTX (10^−8^ mol/L, an α7 nAChR specific antagonist), for 30 min before nicotine (10^−5^ mol/L, 12 h) stimulation, and examined autophagy related protein and autolysosomes expression. The autophagy expression of hPDLCs was detected by immunofluorescence, Western blot and TEM. According to immunofluorescence results (Fig. 2A, B), nicotine treatment of hPDLCs significantly increased LC3 fluorescence puncta (*p* < 0.0001). Compared with the nicotine group, the LC3 fluorescence puncta in nicotine combined with α-BTX treatment group were significantly reduced (*p* < 0.001). There was no significant difference in the number of LC3 fluorescent puncta between the α-BTX alone group and the control group. According to Western blot (Fig. 2C- E) and TEM (Fig. 2F, G) results, we detected the LC3II and Beclin-1 protein expression and autolysosomes in hPDLCs, and the results were consistent with immunofluorescence. Therefore, α-BTX treatment blocked the regulation of autophagy of hPDLCs by nicotine. This finding indicates that the effects of nicotine on the autophagy of hPDLCs are mediated by α7 nAChR.

### Effects of 3-MA on the expression of nicotine-induced autophagy of hPDLCs

**Fig. 3:**
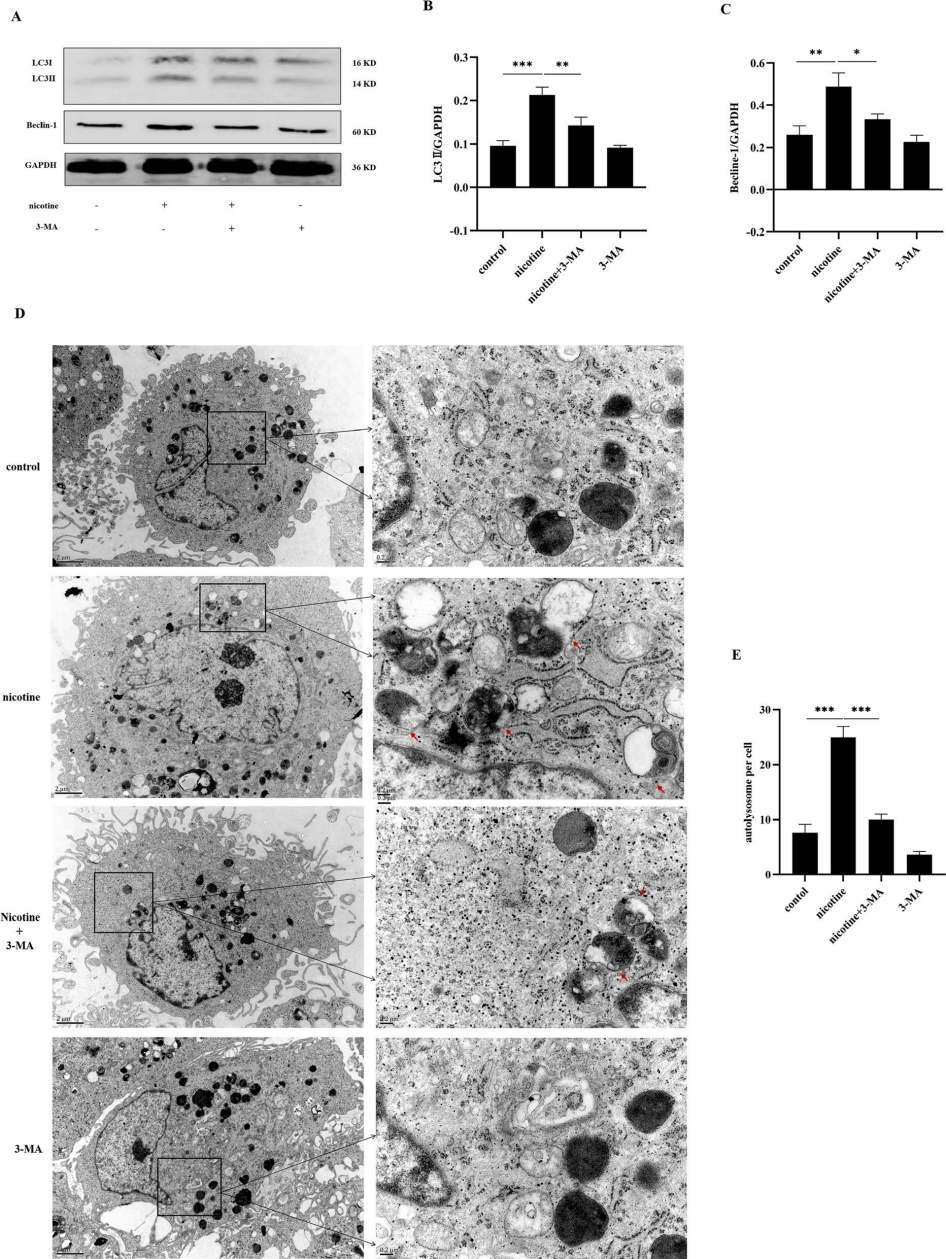
Effects of nicotine and/or 3-MA on the expression of autophagy-related markers in hPDLCs. **A** LC3II and Beclin-1 expression in hPDLCs assessed by Western blot analysis; **B** LC3II protein quantitative analysis. Data were expressed as mean ± SD from at least three independent experiments; **C** Beclin-1 protein quantitative analysis. Data were expressed as mean ± SD from at least three independent experiments; **D** TEM was used to evaluate autophagy induced by nicotine (10^−5^ mol/L, 12 h) and/or α-BTX (10^−8^ mol/L, 12.5 h). Magnification, 40,000×. Scale bar, 0.5 µm. The red arrows represent autolysosome; **E** Quantitative analysis of autolysosomes in one cell, at least 3 cells in each group for statistics (*p < 0.05, **p < 0.005, ***p < 0.001)

To further examine the association between nicotine and autophagy in hPDLCs, we pretreated hPDLCs with 3-MA (10^−3^ mol/L, a PI3K inhibitor that effectively blocks autophagy), for 30 min before nicotine stimulation, and examined autophagy related protein and autolysosomes expression. Western blot results (Fig. 3A–C) showed that nicotine significantly increased the expression of LC3II and Beclin-1 in hPDLCs. Compared with the nicotine group, LC3II and Beclin-1 protein expression in nicotine combined with 3-MA treatment group was significant decreased. We also analyzed the expression of autolysosomes by TEM (Fig. 3D, E), the results also showed a similar trend, indicating that 3-MA can inhibit nicotine-induced autophagy of hPDLCs. It further illustrates the role of PI3K pathway in nicotine-induced autophagy.

### Nicotine may partly depend on the α7 nAChR-PI3K pathway to induce hPDLC to secrete IL-1β and IL-8

**Fig. 4:**
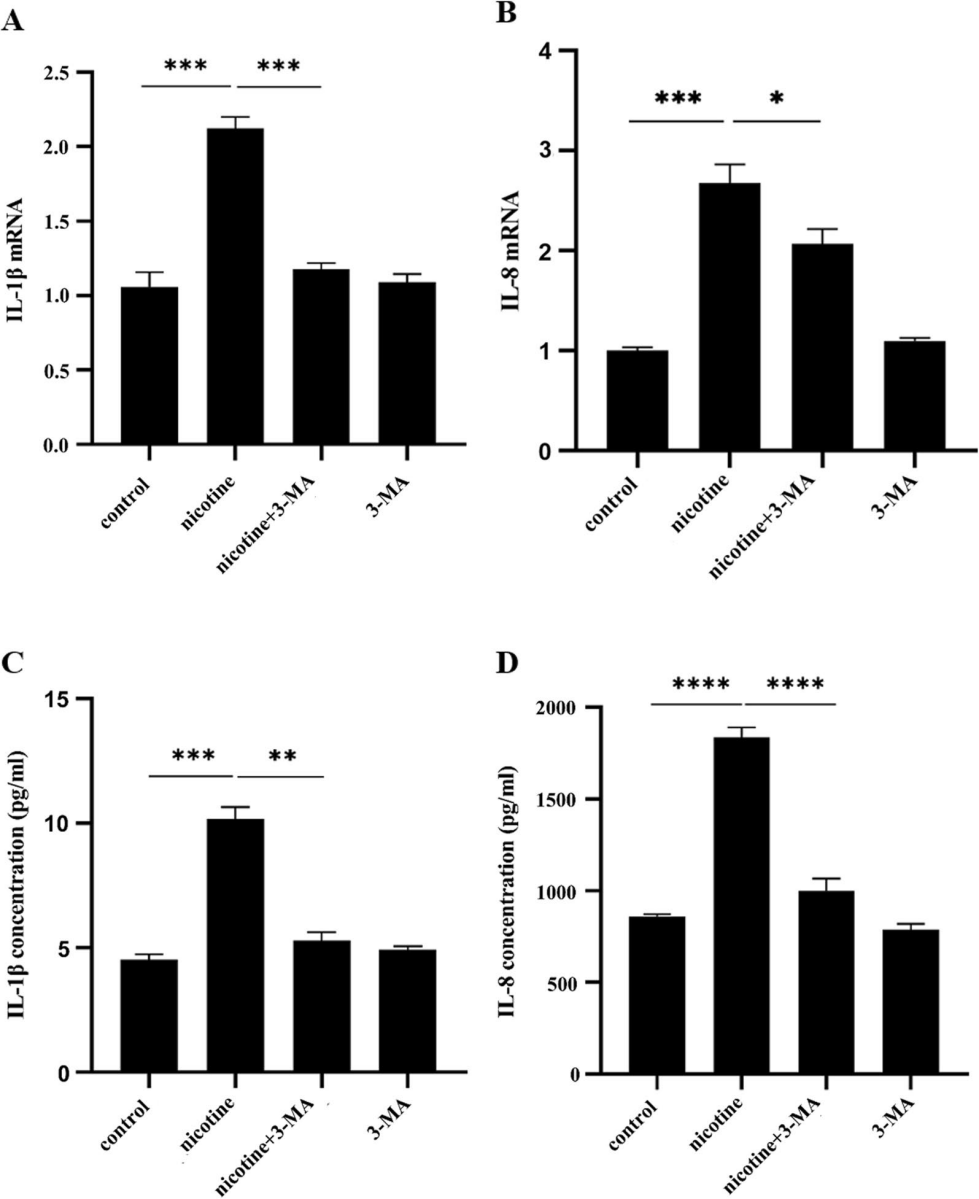
Effects of nicotine and/or 3-MA on the production of IL-1β and IL-8. **A** The relative expression of IL-1β mRNA from hPDLCs after nicotine and/or 3-MA treatment; **B** The relative expression of IL-8 mRNA from hPDLCs after nicotine and/or 3-MA treatment; **C** Release of IL-1β from hPDLCs after nicotine and/or 3-MA application; **D** Release of IL-8 from hPDLCs after nicotine and/or 3-MA application. Data from three independent experiments were presented as mean ± SD (*p < 0.05, **p < 0.005, ***p < 0.001, ****p < 0.0001)

In our previous study, we had repeatedly demonstrated that nicotine induced the production of IL-1β and IL-8 in hPDLCs through α7 nAChR. To further investigate whether autophagy is involved in the secretion of inflammatory cytokines in hPDLCs. RT-qPCR and ELISA were used to detect the expression of inflammatory factors in hPDLCs after nicotine and/or 3-MA treatment. RT-qPCR results indicated that nicotine upregulated IL-1β and IL-8 mRNA expression in hPDLCs (Fig. 4A, B). Compared with the nicotine group, the mRNA expression of IL-1β and IL-8 in nicotine combined with 3-MA treatment group was significantly decreased (*p* < 0.001, *p* < 0.05). IL-1β and IL-8 mRNA expression was not significantly different between the 3-MA alone group and the control group (*p* > 0.05). ELISA results showed that nicotine can significantly promote the secretion of IL-1β and IL-8 from hPDLCs (Fig. 4C, D), Compared with the nicotine group, the secretion of IL-1β and IL-8 in nicotine combined with 3-MA treatment group was significantly decreased (*p* < 0.005, *p* < 0.0001). There was no significant different in the secretion of IL-1β and IL-8 between the 3-MA treatment group and the control group (*p* > 0.05). Collectively, these results indicate that nicotine-induced production of IL-1β and IL-8 from hPDLCs is partially dependent on the α7 nAChR-PI3K pathway.

## Discussion

This study explored the mechanism by which nicotine regulated autophagy of hPDLCs and thereby up-regulated the release of inflammatory factors from hPDLCs. We found that nicotine significantly enhanced the autophagy of hPDLCs and secretion of IL-1β and IL-8 through α7 nAChR thus aggravating the inflammatory response of the periodontal tissues and periodontitis.

Autophagy is an intracellular process that degrades organelles or cellular components in order to ensure the maintenance of cell homeostasis [27]. Autophagy participates in the renewal, pluripotency, differentiation, proliferation, and aging of stem cells [28]. Stem cells of dental origin have gained increasing attention in recent years due to their mesenchymal stem cells (MSC)-like characteristics. Dental pulp stem cells activate autophagy as a pro-survival cytoprotective mechanism in response to hydroxyethyl methacrylate injury [29]. Metformin may prevent cytotoxicity in hPDLCs exposed to Polydopamine-templated hydroxyapatite by reducing ROS via autophagy-related signaling pathways, and may also enhance osteogenic differentiation in hPDLCs [30].

In recent years, studies have found that periodontitis is closely related to autophagy of hPDLCs [31]. Nicotine is the subject toxic component in tobacco and an important risk factor for periodontitis [32]. Preliminary research found nicotine can change the morphology and structure of periodontal tissues cells and cause pathological changes in periodontal tissues [18, 33]. To investigate the role of nicotine in autophagy of hPDLCs, hPDLCs were treated with different concentrations of nicotine for different time. We found nicotine-induced hPDLCs autophagy in a dose-and time-dependent manner (Fig. 1). Consistent with this, nicotine enhanced the autophagy expression in human cancer colon cells and ocular smooth muscle cells, and the autophagy expression showed a nicotine concentration and time-dependent [34, 35]. The results of our in vivo study revealed that nicotine can up-regulate the autophagy expression of hPDLCs.

α7 nAChR is a predominant subunit of nAChRs, as well as a potent target of the nicotine binding receptor [17]. In previous studies, we had confirmed the functional expression of α7 nAChR in PDL tissues and hPDLCs, and nicotine can up-regulate the expression of α7 nAChR [7, 8, 33]. Some existing studies had confirmed the complex connection between nAChRs, nicotine and autophagy in other cell types [34]. Huang et al. [36] reported that nicotine up-regulated the expression of α7 nAChR and autophagy in SH-SY-5Y cells, and α7 nAChR specific antagonist (α-BTX) can inhibit nicotine-induced autophagy. Consistent with this, in this study we observed that α-BTX inhibited nicotine-induced autophagy (Fig. 2), suggesting that α7 nAChR is related to nicotine-induced autophagy of hPDLCs. To our knowledge, this is the first report to prove that nicotine regulates autophagy in hPDLCs via α7 nAChR.

Autophagy plays an important role in the occurrence and development of inflammation and immune response [37]. In recent years, more and more studies in vitro and in vivo have confirmed the role of autophagy in periodontitis [9], but there is no sufficient evidence to confirm whether the role of autophagy in periodontitis is protective or pathological [38]. An et al. [39] reported that autophagy disorder in periodontitis was associated with protection. Increased autophagy was required to protect periodontal ligament stem cells from apoptosis in inflammatory microenvironment. Alternatively, Tsuda et al. [40] revealed that the overexpression of autophagy induced cell death in gingival epithelial cell line, suggesting its pathological involvement in periodontitis. In this study, we found that after using nicotine, the autophagy of hPDLCs was significantly enhanced, and the release of IL-1β and IL-8 was enhanced through autophagy (Fig. 4). This finding suggests that autophagy is related to the pathogenesis of smoking-related periodontitis.

Previously, we had repeatedly demonstrated nicotine induced the production of IL-1β and IL-8 via α7 nAChR in hPDLCs [6, 8]. Studies found that nicotine up-regulated the expression of IL-8 and IL-1β in human gingival epithelial cells through the nAChR pathway [41]. IL-1β is not only related to smoking-related periodontitis, but increasing IL-1β can trigger a series of inflammatory reactions and promote bone resorption [42]. IL-8 is associated with periodontal status, the level of IL-8 in gingival crevicular fluid is valuable in detecting the inflammation of periodontal tissue [43]. It is speculated that IL-1β and IL-8 could be potential therapeutic targets for smoking-related periodontitis.

Autophagy plays a role in determining the fate of IL-1β and IL-8 [44, 45]. Starved macrophages during inflammasome activation have been reported to secrete IL-1β in an autophagy-dependent manner [46]. Furthermore, intracellular IL-1β colocalized with LC3 puncta indicating the intersection between the autophagy process and secretion of IL-1β [47]. Autophagy was required for toll-like receptor-mediated IL-8 production in intestinal epithelial cells [48]. ATG can promote the release of IL-8 in human airway epithelial cells, contributing to neutrophilic airway inflammation in the pathogenesis of adult asthma [49]. In our research, we found that nicotine up-regulated the expression of LC3II, Becline-1 protein and the secretion of inflammatory factors IL-1β and IL-8. This result can be inhibited by 3-MA, which is a PI3K inhibitor that can effectively block autophagy (Figs. 3 and 4). PI3K is a complex signaling system. We preliminarily confirmed that the a7 nAChR/PI3K pathway can activate autophagy by nicotine and promote the secretion of inflammatory cytokines IL-1β and IL-8. The specific molecular mechanism remains to be further studied.

In summary, nicotine regulated the autophagy level of hPDLCs through α7 nAChR, and significantly enhanced the secretion of IL-1β and IL-8, thereby aggravating the inflammatory response of hPDLCs. Although we reported interesting findings, these results were based on the response of certain cells in vitro, rather than mimicking in vivo or clinical conditions. Animal models are needed for further studies to confirm the results of this study. In addition, further research should be conducted to explore the detailed molecular mechanisms involving nicotine-α7 nAChR-autophagy pathway and smoking-related periodontitis.

## Conclusions

Nicotine regulated autophagy of hPDLCs through α7 nAChR and in turn the regulation of the release of inflammatory factors IL-1β and IL-8 by hPDLCs. This study provides experimental evidence for the pathological development of smoking-related periodontitis and sheds new light on developing smoking related periodontitis.

## Supplementary Information

**Additional file 1.** The original version of western blot images in Figure 2 and Figure 3.

## Data Availability

The datasets used and/or analyzed during the current study are available from the corresponding author on reasonable requests.

## Abbreviations

hPDLCs: Human periodontal ligament cells
α7 nAChR: Alpha7 nicotinic acetylcholine receptor
α-BTX: Alpha-bungarotoxin
3-MA: 3-Methyladenine
ELISA: Enzyme-linked immunosorbent assay
TEM: Transmission electron microscopy
PDL: Periodontal ligament
FCS: Fetal calf serum

## References

[1] Sanz M, Marco Del Castillo A, Jepsen S, Gonzalez-Juanatey JR, D'Aiuto F, Bouchard P, Chapple I, Dietrich T, Gotsman I, Graziani F (2020). Periodontitis and cardiovascular diseases: Consensus report. J Clin Periodontol.

[2] Kassebaum NJ, Smith AGC, Bernabé E, Fleming TD, Reynolds AE, Vos T, Murray CJL, Marcenes W (2017). Global, regional, and national prevalence, incidence, and disability-adjusted life years for oral conditions for 195 Countries, 1990–2015: a systematic analysis for the global burden of diseases, injuries, and risk factors. J Dent Res.

[3] Leite FRM, Nascimento GG, Baake S, Pedersen LD, Scheutz F, López R (2019). Impact of Smoking Cessation on Periodontitis: A Systematic Review and Meta-analysis of Prospective Longitudinal Observational and Interventional Studies. Nicotine Tobacco Res.

[4] Carson SJ, Burns J (2016). Impact of smoking on tooth loss in adults. Evid Based Dent.

[5] Takeuchi-Igarashi H, Kubota S, Tachibana T, Murakashi E, Takigawa M, Okabe M, Numabe Y (2016). Matrix remodeling response of human periodontal tissue cells toward fibrosis upon nicotine exposure. Odontology.

[6] Wu LZ, Duan DM, Liu YF, Ge X, Zhou ZF, Wang XJ (2013). Nicotine favors osteoclastogenesis in human periodontal ligament cells co-cultured with CD4(+) T cells by upregulating IL-1β. Int J Mol Med.

[7] Zhou Z, Liu F, Wang L, Zhu B, Chen Y, Yu Y, Wang X (2020). Inflammation has synergistic effect with nicotine in periodontitis by up-regulating the expression of α7 nAChR via phosphorylated GSK-3β. J Cell Mol Med.

[8] Wu L, Zhou Y, Zhou Z, Liu Y, Bai Y, Xing X, Wang X (2014). Nicotine induces the production of IL-1β and IL-8 via the α7 nAChR/NF-κB pathway in human periodontal ligament cells: an in vitro study. Cell Physiol Biochem.

[9] Greabu M, Giampieri F, Imre MM, Mohora M, Totan A, Pituru SM, Ionescu E (2020). Autophagy, one of the main steps in periodontitis pathogenesis and evolution. Molecules.

[10] Pugsley HR (2017). Quantifying autophagy: measuring LC3 puncta and autolysosome formation in cells using multispectral imaging flow cytometry. Methods.

[11] Ni HM, Bockus A, Wozniak AL, Jones K, Weinman S, Yin XM, Ding WX (2011). Dissecting the dynamic turnover of GFP-LC3 in the autolysosome. Autophagy.

[12] Mizushima N, Levine B (2020). Autophagy in human diseases. N Engl J Med.

[13] Bullon P, Cordero MD, Quiles JL, Ramirez-Tortosa Mdel C, Gonzalez-Alonso A, Alfonsi S, García-Marín R, de Miguel M, Battino M (2012). Autophagy in periodontitis patients and gingival fibroblasts: unraveling the link between chronic diseases and inflammation. BMC Med.

[14] Mei YM, Li L, Wang XQ, Zhang M, Zhu LF, Fu YW, Xu Y (2020). AGEs induces apoptosis and autophagy via reactive oxygen species in human periodontal ligament cells. J Cell Biochem.

[15] Xing R, Cheng X, Qi Y, Tian X, Yan C, Liu D, Han Y (2020). Low-dose nicotine promotes autophagy of cardiomyocytes by upregulating HO-1 expression. Biochem Biophys Res Commun.

[16] Wang Z, Liu B, Zhu J, Wang D, Wang Y (2019). Nicotine-mediated autophagy of vascular smooth muscle cell accelerates atherosclerosis via nAChRs/ROS/NF-κB signaling pathway. Atherosclerosis.

[17] Wu L, Yang K, Gui Y, Wang X (2020). Nicotine-upregulated miR-30a arrests cell cycle in G1 phase by directly targeting CCNE2 in human periodontal ligament cells. Biochem Cell Biol.

[18] Zhou Z, Li B, Dong Z, Liu F, Zhang Y, Yu Y, Shang F, Wu L, Wang X, Jin Y (2013). Nicotine deteriorates the osteogenic differentiation of periodontal ligament stem cells through α7 nicotinic acetylcholine receptor regulating Wnt pathway. PLOS ONE.

[19] Liu D, Yang Y, Liu Q, Wang J (2011). Inhibition of autophagy by 3-MA potentiates cisplatin-induced apoptosis in esophageal squamous cell carcinoma cells. Med Oncol (Northwood, London, England).

[20] Wu YT, Tan HL, Shui G, Bauvy C, Huang Q, Wenk MR, Ong CN, Codogno P, Shen HM (2010). Dual role of 3-methyladenine in modulation of autophagy via different temporal patterns of inhibition on class I and III phosphoinositide 3-kinase. J Biol Chem.

[21] Hahnvajanawong C, Sahakulboonyarak T, Boonmars T, Reutrakul V, Kerdsin A, Boueroy P (2021). Inhibitory effect of isomorellin on cholangiocarcinoma cells via suppression of NF-κB translocation, the phosphorylated p38 MAPK pathway and MMP-2 and uPA expression. Exp Ther Med.

[22] Wei W, An Y, An Y, Fei D, Wang Q (2018). Activation of autophagy in periodontal ligament mesenchymal stem cells promotes angiogenesis in periodontitis. J Periodontol.

[23] Zhang Y, Zhang Y, Jin XF, Zhou XH, Dong XH, Yu WT, Gao WJ (2019). The role of astragaloside IV against cerebral ischemia/reperfusion injury: suppression of apoptosis via promotion of P62-LC3-autophagy. Molecules.

[24] Zhang S, Li Y, Tu Y (2021). Lidocaine attenuates CFA-induced inflammatory pain in rats by regulating the MAPK/ERK/NF-κB signaling pathway. Exp Ther Med.

[25] Livak KJ, Schmittgen TD (2001). Analysis of relative gene expression data using real-time quantitative PCR and the 2(-Delta Delta C(T)) method. Methods.

[26] Ge X, Liu YF, Wong Y, Wu LZ, Tan L, Liu F, Wang XJ (2016). Impact of nicotine on the interplay between human periodontal ligament cells and CD4+ T cells. Hum Exp Toxicol.

[27] Yu L, Chen Y, Tooze SA (2018). Autophagy pathway: cellular and molecular mechanisms. Autophagy.

[28] Babaei G, Aziz SG, Jaghi NZZ (2021). EMT, cancer stem cells and autophagy; The three main axes of metastasis. Biomed Pharmacother.

[29] Diomede F, Tripodi D, Trubiani O, Pizzicannella J (2019). HEMA Effects on autophagy mechanism in human dental pulp stem cells. Materials.

[30] Yang Z, Gao X, Zhou M, Kuang Y, Xiang M, Li J, Song J (2019). Effect of metformin on human periodontal ligament stem cells cultured with polydopamine-templated hydroxyapatite. Eur J Oral Sci.

[31] Zhang X, Jin Y, Wang Q, Jian F, Li M, Long H, Lai W (2020). Autophagy-mediated regulation patterns contribute to the alterations of the immune microenvironment in periodontitis. Aging.

[32] Isik Andrikopoulos G, Farsalinos K, Poulas K (2019). Electronic nicotine delivery systems (ENDS) and their relevance in oral health. Toxics.

[33] Wang XJ, Liu YF, Wang QY, Tsuruoka M, Ohta K, Wu SX, Yakushiji M, Inoue T (2010). Functional expression of alpha 7 nicotinic acetylcholine receptors in human periodontal ligament fibroblasts and rat periodontal tissues. Cell Tissue Res.

[34] Yan HY, Wen X, Chen LZ, Feng YT, Liu HX, Qu W, Zhao WH, Xu DQ, Ping J (2021). Augmented autophagy suppresses thymocytes development via Bcl10/p-p65 pathway in prenatal nicotine exposed fetal mice. Ecotoxicol Environ Saf.

[35] Pelissier-Rota MA, Pelosi L, Meresse P, Jacquier-Sarlin MR (2015). Nicotine-induced cellular stresses and autophagy in human cancer colon cells: a supportive effect on cell homeostasis via up-regulation of Cox-2 and PGE(2) production. Int J Biochem Cell Biol.

[36] Hung SY, Huang WP, Liou HC, Fu WM (2009). Autophagy protects neuron from Abeta-induced cytotoxicity. Autophagy.

[37] Deretic V, Levine B (2018). Autophagy balances inflammation in innate immunity. Autophagy.

[38] Yang Y, Huang Y, Li W (2021). Autophagy and its significance in periodontal disease. J Periodontal Res.

[39] An Y, Liu W, Xue P, Zhang Y, Wang Q, Jin Y (2016). Increased autophagy is required to protect periodontal ligament stem cells from apoptosis in inflammatory microenvironment. J Clin Periodontol.

[40] Tsuda H, Ochiai K, Suzuki N, Otsuka K (2010). Butyrate, a bacterial metabolite, induces apoptosis and autophagic cell death in gingival epithelial cells. J Periodontal Res.

[41] Kashiwagi Y, Yanagita M, Kojima Y, Shimabukuro Y, Murakami S (2012). Nicotine up-regulates IL-8 expression in human gingival epithelial cells following stimulation with IL-1β or *P. gingivalis* lipopolysaccharide via nicotinic acetylcholine receptor signalling. Arch Oral Biol.

[42] Cheng R, Wu Z, Li M, Shao M, Hu T (2020). Interleukin-1β is a potential therapeutic target for periodontitis: a narrative review. Int J Oral Sci.

[43] Lagdive SS, Marawar PP, Byakod G, Lagdive SB (2013). Evaluation and comparison of interleukin-8 (IL-8) level in gingival crevicular fluid in health and severity of periodontal disease: a clinico-biochemical study. Indian J Dent Res.

[44] Claude-Taupin A, Bissa B, Jia J, Gu Y, Deretic V (2018). Role of autophagy in IL-1β export and release from cells. Semin Cell Dev Biol.

[45] Korhonen E, Piippo N, Hytti M, Hyttinen JMT, Kaarniranta K, Kauppinen A (2019). SQSTM1/p62 regulates the production of IL-8 and MCP-1 in IL-1β-stimulated human retinal pigment epithelial cells. Cytokine.

[46] Torp MK, Yang K, Ranheim T, Husø Lauritzen K, Alfsnes K, Vinge LE, Aukrust P, Stensløkken KO, Yndestad A, Sandanger Ø (2019). Mammalian target of Rapamycin (mTOR) and the proteasome attenuates IL-1β expression in primary mouse cardiac fibroblasts. Front Immunol.

[47] Cadwell K (2016). Crosstalk between autophagy and inflammatory signalling pathways: balancing defence and homeostasis. Nat Rev Immunol.

[48] Li YY, Ishihara S, Aziz MM, Oka A, Kusunoki R, Tada Y, Yuki T, Amano Y, Ansary MU, Kinoshita Y (2011). Autophagy is required for toll-like receptor-mediated interleukin-8 production in intestinal epithelial cells. Int J Mol Med.

[49] Pham DL, Kim SH, Losol P, Yang EM, Shin YS, Ye YM, Park HS (2016). Association of autophagy related gene polymorphisms with neutrophilic airway inflammation in adult asthma. Korean J Intern Med.

