# Supplementary figures and images for: Nicotine regulates autophagy of human periodontal ligament cells through α7 nAchR that promotes secretion of inflammatory factors IL-1β and IL-8
Source: BMC Oral Health. 2021 Nov 3;21:560. doi: 10.1186/s12903-021-01894-5 (PMC8565023; doi:10.1186/s12903-021-01894-5)

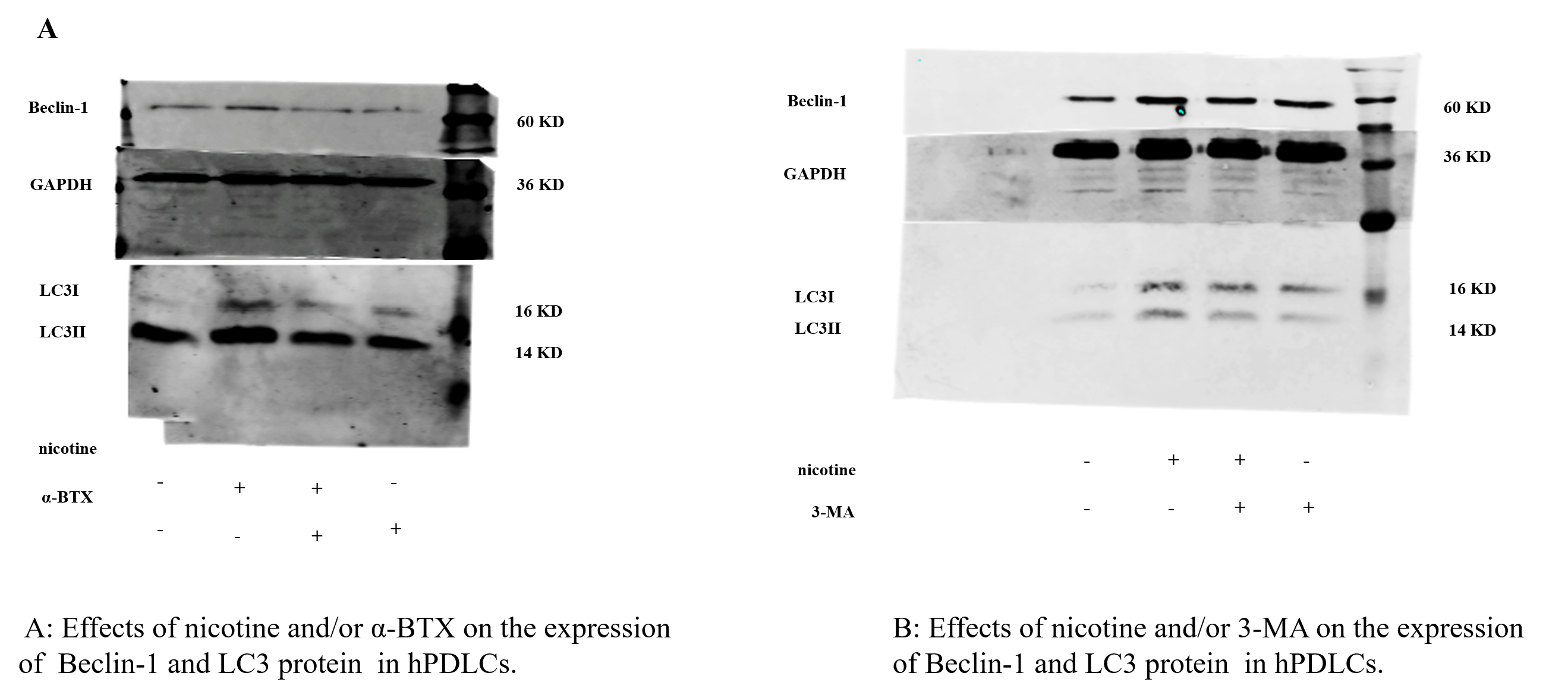

Supplement: Supplementary file 1 — Additional file 1. The original version of western blot images in Figure 2 and Figure 3. [file 12903_2021_1894_MOESM1_ESM.tif]
